# Obesity as a risk factor for multimorbidity: a 23-years population-based prospective study

**DOI:** 10.1177/26335565261478101

**Published:** 2026-08-17

**Authors:** Jussa-Pekka Välitalo, Paula Pesonen, Jouko Saramies, Sirkka Keinänen-Kiukaanniemi, Hannu Uusitalo, Julia Lybeck, Esko Hussi, Kadri Suija, Jaakko Tuomilehto, Juha Auvinen

**Affiliations:** 1 Research Unit of Population Health, 6370 University of Oulu, Oulu, Finland; 2 Medical Research Center, Oulu University Hospital, Oulu, Finland; 3 Infrastructure for Population Studies, Faculty of Medicine, 6370 University of Oulu, Oulu, Finland; 4 South Karelia Wellbeing Services County, Savitaipale, Finland; 5 North Ostrobothnia Wellbeing Services County, Pyhäjärvi, Finland; 6 SILK, Department of Ophthalmology, Faculty of Medicine and Health Technology, 12520 Tampere University, Tampere, Finland; 7 Tays Eye Centre, Tampere University Hospital, Tampere, Finland; 8 Institute of Family Medicine and Public Health, 337108 University of Tartu, Tartu, Estonia; 9 South Karelia Wellbeing Services County, Lappeenranta, Finland; 10 Institute of Public Health and Clinical Nutrition, Faculty of Health Sciences, University of Eastern Finland, Kuopio, Finland; 11 Department of Public Health, 3835 University of Helsinki, Helsinki, Finland

**Keywords:** body mass index, body roundness index, waist circumference, multimorbidity, obesity

## Abstract

**Background:**

Obesity and multimorbidity are increasing challenges for public health. While obesity is a well-recognized risk factor for multimorbidity, evidence commonly comes from cross-sectional studies using body mass index (BMI). Evidence on the body roundness index (BRI), a novel indicator of central obesity, remains limited.

**Objectives:**

This study assessed midlife obesity as a risk factor for multimorbidity in a Finnish population cohort during 23 years of follow-up.

**Methods:**

A total of 1007 participants born between 1933 and 1956 and living in the Savitaipale municipality were enrolled between 1994 and 1996. Baseline data were collected using questionnaires and clinical examinations. Information on 38 chronic diseases was obtained from health registers. Obesity was assessed using BMI, waist circumference (WC), and BRI. Cox proportional hazards models estimated associations with incident multimorbidity, and receiver operating characteristic (ROC) analysis was used to derive BMI, WC and BRI thresholds with the best sensitivity–specificity balance.

**Results:**

Over 23 years, obesity defined by BMI and WC, as well as higher BRI, was associated with increased multimorbidity risk. Hazard ratios ranged from 1.52 (95% CI: 1.21–1.92) to 2.17 (95% CI: 1.59–2.97) in women and from 2.41 (95% CI: 1.82–3.19) to 2.79 (95% CI: 2.00–3.90) in men, depending on obesity and multimorbidity definitions. ROC-derived thresholds for multimorbidity prediction were: BMI 26.4 in women and 26.1 in men; WC 76.3 cm and 93.3 cm; and BRI 3.41 and 3.70, respectively.

**Conclusion:**

This 23-year study highlights midlife obesity as a risk factor for multimorbidity in later life.

## Introduction

Multimorbidity is commonly defined, in both research and clinical guidelines, as the coexistence of two or more long-term conditions within the same individual.^1–4^ Multimorbidity is associated with increased mortality,^5^ higher health care costs,^6^ greater use of both primary and secondary care,^7^ and reduced quality of life.^8–10^ Prevalence of multimorbidity increases with age,^11,12^ posing a growing challenge for both individuals and health care systems. The estimated global prevalence of multimorbidity among adults is 37.2% (95% CI 34.9–39.4%), and in Europe 39.2% (95% CI 33.2–45.2%).^12^

According to the WHO, one in eight people worldwide had obesity defined by body mass index (BMI) ≥30 kg/m2, in 2022.^13^ In Finland, it is estimated that 24% of women and 30% of men may have obesity by 2060, with similar estimates suggested for other Nordic countries (Iceland, Denmark, Norway and Sweden).^14^

Obesity has been associated with multimorbidity in several cross-sectional studies^15–18^ and according to a recent meta-analysis of longitudinal studies.^19^ However, the meta-analysis found only two publications with follow-up periods longer than 20 years.^20,21^ Both studies included only women and defined multimorbidity using fewer than 12 chronic diseases, although Fortin and coworkers recommended at least 12 diagnoses in such calculations.^22^ Obesity is commonly considered as a risk factor for multimorbidity using body mass index (BMI) and waist circumference (WC),^19,23–25^ whereas studies incorporating the body roundness index (BRI), an anthropometric measure derived from height and WC and proposed to reflect visceral adiposity,^26^ remain rare.^27^ Although BRI is associated with cardiometabolic multimorbidity,^28,29^ its application in multimorbidity research beyond cardiometabolic diseases is still limited.

This study aimed to evaluate the role of midlife obesity as a risk factor for developing multimorbidity in a Finnish population cohort over a 23-year follow-up. To account for the heterogeneity of obesity, we selected three anthropometric measures reflecting different aspects of adiposity. BMI was included as a widely used indicator of general obesity, although it does not account for body fat distribution or body composition.^30^ WC was included as an established measure of central obesity.^30^ In addition, BRI was incorporated as a shape-based measure reflecting abdominal and visceral adiposity.^26^ The present study, for the first time to the authors’ knowledge, also aimed to explore sex-specific BRI thresholds for multimorbidity risk assessment and to identify cut-off values that optimize predictive performance in terms of sensitivity and specificity.

Although obesity could be included among the conditions counted to define multimorbidity, we treated obesity as a risk factor. Obesity is widely regarded as one of the leading modifiable risk factors in public-health frameworks,^31^ highlighting the need to understand the long-term consequences and risks of obesity for multimorbidity. Reducing obesity in midlife may therefore have meaningful implications at both the individual and population levels for preventing multimorbidity later in life.

## Methods

### Study design and study population

This longitudinal cohort study is based on the Savitaipale Study, conducted in the rural municipality of Savitaipale in Finland. The design of the Savitaipale Study, the characteristics of the study population, and the 10- and 22-year follow-up examinations have been described in detail previously by Saramies et al.^32^

At baseline (1994–1996), the target population consisted of all 1561 individuals born between 1933 and 1956 who were registered as residents of Savitaipale on 27 May 1996. A total of 1168 individuals participated in the study at baseline (77.5% of those invited) and all clinical examinations were performed at the local primary health care centre. The present multimorbidity study represents a secondary data analysis of the data collected in the Savitaipale Study, supplemented with linked routinely collected healthcare data. The original purpose of the Savitaipale Study at baseline was to screen for and enable early detection of diabetes; therefore, individuals with known type 1 diabetes at baseline (*n* = 2), and those with missing height or weight data (*n* = 3), were excluded. To ensure participant confidentiality, those classified as underweight based on BMI (*n* = 5) were excluded from further analyses due to the very small group size. In addition, participants who did not consent to linkage with health registry data were excluded, resulting in a final analytical sample of 1007 participants for this study. Baseline characteristics are presented in Table 1.

**Table 1.** Baseline characteristics of the study population.

​	Total	Women	Men
**Number of participants**	1007	506	501
**Age: mean years (SD)**	52.8 (7.2)	52.7 (7.1)	52.8 (7.2)
**Number of chronic diseases at baseline**			
0 diseases, *n* (%)	630 (62.6)	319 (63.0)	311 (62.1)
1 disease, *n* (%)	227 (22.5)	122 (24.1)	105 (21.0)
≥2 diseases, *n* (%)	150 (14.9)	65 (12.8)	85 (17.0)
**BMI at baseline, kg/m** ^**2**^			
18.5-24.9, *n* (%)	403 (40.0)	212 (41.9)	191 (38.1)
25-29.9, *n* (%)	424 (42.1)	193 (38.1)	231 (46.1)
≥30, *n* (%)	180 (17.9)	101 (20.0)	79 (15.8)
**WC at baseline, cm**			
Women <80, men <94, *n* (%)	N/A	177 (35.0)	254 (50.7)
Women 80-87.9, men 94-101.9, *n* (%)	N/A	130 (25.7)	127 (25.3)
Women ≥88, men ≥102, *n* (%)	N/A	195 (38.5)	119 (23.8)
Waist circumference data missing, *n* (%)	5 (0.5)	4 (0.8)	1 (0.2)
**BRI at baseline**			
Mean BRI values (SD)	N/A	4.06 (1.66)	4.27 (1.35)
BRI data missing, *n* (%)	5 (0.5)	4 (1.4)	1 (0.2)
**Smoking status at baseline**			
Never smoker, *n* (%)	533 (52.9)	365 (72.1)	168 (33.5)
Ex-smoker or current smoker, *n* (%)	454 (45.1)	130 (25.7)	324 (64.7)
Smoking data missing, *n* (%)	20 (2.0)	11 (2.2)	9 (1.8)

SD: Standard deviation, BMI: Body mass index, WC: Waist circumference BRI: Body roundness index N/A: not applicable.

The study was conducted in accordance with the Declaration of Helsinki and approved by the Ethics Review Board of the South Karelia Hospital District (approval number 24/98).

### Definition of multimorbidity

Multimorbidity was defined as the coexistence of two or more chronic diseases as previously established. In addition, thresholds of three or more and four or more chronic diseases were used to model more complex forms of multimorbidity. Information on chronic disease diagnoses and the first recorded occurrence of each diagnosis was obtained from local health care records and national health registers, covering both primary and secondary care.

National registers used in this study include the Register of Primary Health Care Visits, the Hospital Discharge Register (later the Care Register for Health Care), the Drug Reimbursement Register, and the Drug Prescription Purchase Register. These nationwide data collections are statutory, maintained by the Finnish Institute for Health and Welfare (THL), and health-care providers are legally obligated to submit data to THL. The Hospital Discharge Register has been in continuous operation since 1969, and from 1994 onwards as the Care Register for Health Care, enabling comprehensive national coverage and record linkage from secondary care via the personal identification number.

The diagnosis validity in the Finnish patient registry is high for many conditions with diagnosis-specific variation; completeness in the national registries is strongest for hospital-managed diseases (e.g., acute myocardial infarction) and lower for primary-care conditions.^33,34^ Alongside the national secondary care registers and Register of Primary Health Care visits (years 2011-2019), we included local primary care health care data (1996–2010) to improve ascertainment of conditions diagnosed and managed solely in primary care. Secondary health-care diagnoses from the local hospital register were also included from 2005 onwards.

Several approaches exist for selecting chronic diseases to operationalise multimorbidity, and choices have varied depending on the study context and population. In this study, the disease set was defined based on published recommendations and expert deliberation within the study team. Consistent with recommendations for multimorbidity research, we selected common chronic conditions that typically require prolonged care or treatment within health care services.^35–37^

A previous Delphi consensus study on multimorbidity measurement has developed core sets of chronic conditions to include in multimorbidity research through an iterative process involving both professional and public panels.^38^ These panels proposed and refined lists of conditions to reach consensus for research purposes. The list of diagnoses used in this study was adapted from this Delphi framework and modified to ensure its applicability to the study population, as also recommended.^38^ In total, 38 chronic conditions were included. All diagnoses and corresponding ICD-10, ICD-9 and ATC codes are detailed in Supplementary Table 1.

### Definition of obesity

Body weight, height and WC were measured at baseline. Weight was measured using a Nordica 6210 floor scale, with an accuracy of 100 g. Height was measured to the nearest 0.5 cm with a calibrated wall-mounted ruler. WC was measured with a flexible tape ruler at the midpoint between the iliac crest and the lower margins of the rib.

Baseline BMI was calculated (weight (kg)/height^2^ (m^2^)) and divided into three categories: normal weight (BMI 18.5-24.9), overweight (BMI 25-29.9) and obesity (BMI ≥30) according to WHO definitions.^30^ Participants with BMI <18.5 kg/m^2^ (n = 5) were excluded from the study population due to confidentiality considerations.

WC <80 cm was considered normal, 80-87.9 cm overweight, and ≥88 cm obesity for women, and <94 cm, 94-101.9 cm and ≥102 cm for men according to the International Diabetes Federation (IDF).^39^

Body-roundness index (BRI) was calculated with the following formula^26^: BRI = 364.2 − 365.5 × **√** (1 − [WC /2**ℼ**]^2^/[0.5 × height]^2^) where WC and height were measured in centimetres. As no direct cut-off points exist for the BRI, we used sex-specific tertiles.

### Smoking

Smoking is a well-known risk-factor for many diseases.^40^ Smoking status was self-reported at baseline and classified into two categories based on lifetime exposure: never-smokers and ever-smokers (combining former and current smokers). Since distinguishing cumulative exposure between former and current smokers would have been imprecise in this cohort, a two-category classification was considered the most robust approach.^41^

### Statistical analysis

The number of chronic diseases at baseline was described using frequencies and means with standard deviations. Cox regression models were used to calculate the hazard ratios (HR) and 95% confidence intervals (CI) separately for BRI, BMI and WC. The end points in the Cox regression models were ≥2 diseases, ≥3 diseases and ≥4 diseases and the corresponding censored categories were “0-1 diseases”, “0-2 diseases”, and “0-3 diseases”, respectively. Follow-up time was calculated from birth until the endpoint, defined as reaching a given number of diagnosed chronic diseases, death, or the end of follow-up (31 December 2019). Cox regression models were adjusted for baseline age and smoking status and were conducted separately for men and women to account for sex-specific differences. Only subjects with complete covariate data are used in Cox regression models.

Socioeconomic factors have been associated with an increased risk of multimorbidity and may therefore act as a potential confounder of the association between obesity and multimorbidity.^42^ Information on socioeconomic status (SES) and educational level was only available for a subset of participants. SES was categorized into five groups (entrepreneurs, white-collar employees, blue-collar employees, workers, and others), and education into three levels (basic, vocational, and academic). Sensitivity analyses were conducted in these restricted samples to assess potential socioeconomic confounding. Models were additionally adjusted for SES in the subset with available SES data (men: *n* = 182, women: *n* = 212) and, separately, for educational level in the subset with available educational data (men: *n* = 207, women: *n* = 243).

Sankey-diagrams were used to illustrate the proportions and transitions from age 40 years to the end of follow-up, in five-year periods, showing movement from non-multimorbid (0-1 disease) to multimorbid (2 diseases) and more complex multimorbid groups (≥3 diseases) and deaths across different BMI categories. To protect participant privacy, diseases affecting two or fewer individuals were reported as “fewer than three” instead of the exact number.

Receiver operating characteristic (ROC) analysis was conducted to identify optimal cut-off points in continuous baseline anthropometric measures (BMI, WC, and BRI) for predicting later multimorbidity. The aim was to determine thresholds that maximized both sensitivity and specificity, following established methodological principles for ROC analysis.^43^

The statistical analyses were done using RStudio 2023.06.2 and IBM SPSS Statistics for Windows, Version 29 (IBM Corp., Armonk, NY, USA).

Reporting follows the Strengthening the Reporting of Observational Studies in Epidemiology (STROBE) guidelines, and the corresponding STROBE checklist is provided in the Supplementary table S3.

## Results

At study baseline in 1994–1996, the study population was aged between 41–66 years, with a mean age of 52.8 years (SD 7.2). The prevalence of multimorbidity (≥2 chronic diseases) was 12.8% (*n =* 65*)* among women and 17.1% (*n =* 85) among men (Table 1). By the end of the 23-year follow-up, 87.0% (*n =* 440*)* of women and 88.3% (*n =* 444*)* of men had been diagnosed with ≥2 chronic diseases.

At baseline, 20.0% (*n* = 101) of women and 15.8% (*n =* 79) of men were classified as having obesity based on BMI. When defined by WC, 38.5% (n = 195) of women and 23.8% (*n* = 119) of men had abdominal obesity. The cut-off values for BRI tertiles were 3.13 and 4.43 for women and 3.59 and 4.47 for men.

At baseline, the prevalence of multimorbidity (≥2 diseases) among participants with normal BMI was 8.0% (n = 17) in women and 14.0% (n = 27) in men, compared with 20.8% (*n* = 21) in women and 35.4% (*n* = 28) in men among those with obesity (BMI ≥30).

Similarly, multimorbidity was present in 9.6% (n = 17) of women and 11.4% (n = 29) of men with normal waist circumference, increasing to 19.0% (n = 37) among women and 34.5% (n = 41) among men with obesity defined by waist circumference.

Baseline prevalences of multimorbidity across tertiles of BRI among women were 9.0% (*n* = 15), 11.3% (*n* = 19), and 17.9% (*n* = 30), and among men, 13.3% (*n* = 22), 10.8% (*n* = 18), and 26.8% (*n* = 45).

**Figure 1.**
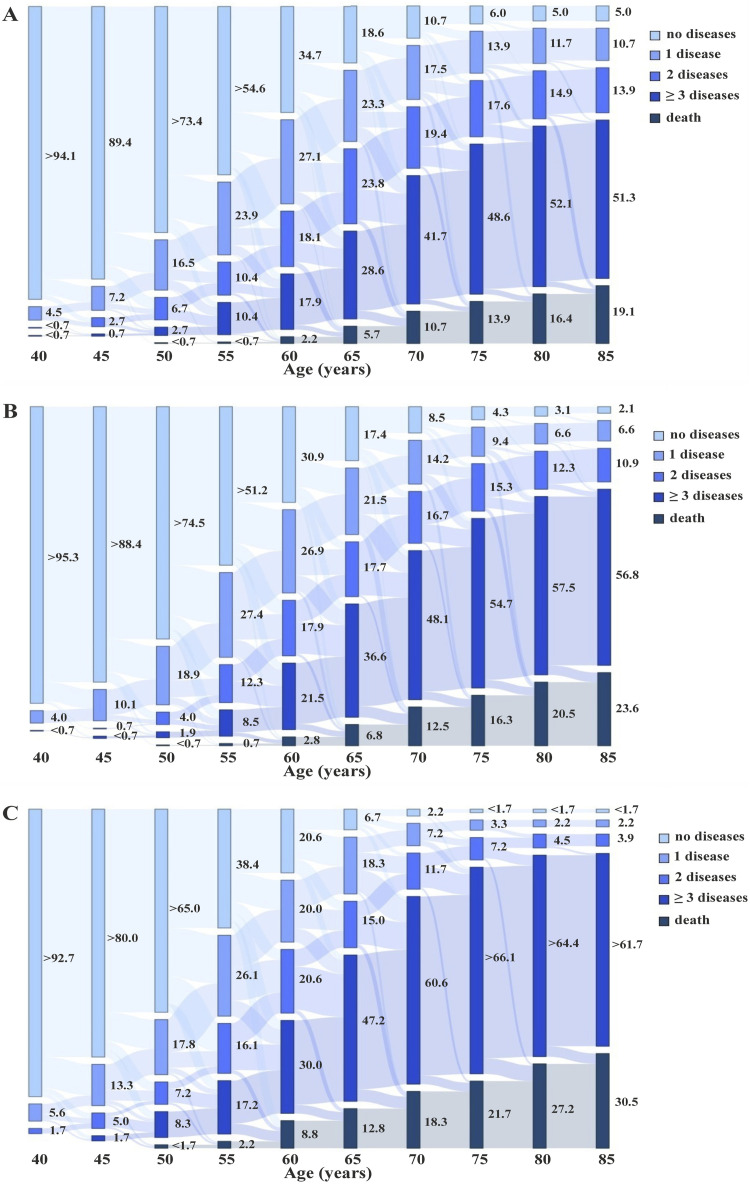
Prevalence (%) of the number of diagnosed chronic diseases in the different age and different baseline BMI classes visualized using Sankey diagrams. Figure 1A participants with BMI 18.5-24.9, 1B participants with BMI 25-29.9, and 1C participants with BMI ≥30.

Figure 1(a)–(c) illustrates the prevalence and transitions between categories of chronic disease counts across age groups, as visualized using Sankey diagrams. Until the end of follow-up, the three most common chronic diseases among women were hypertension (58.9%, *n* = 298), osteoarthritis (49.0%, *n* = 248), and ischemic heart diseases (31.2%, *n* = 158), and among men hypertension (52.7%, *n* = 264), prostate hyperplasia (42.9%, *n* = 215), and ischemic heart diseases (32.5%, *n* = 163) (Supplementary Figures 1A and 1B).

Table 2 presents the occurrence of multimorbidity over a 23-year follow-up by baseline BMI, WC and BRI, using Cox regression analysis adjusted for baseline age and smoking status. Women and men with BMI ≥30 at baseline had a 1.75–2.17-fold (95% CI: 1.35–2.97) and 2.41–2.79-fold (95 % CI: 1.82–3.90) risk of multimorbidity, respectively, compared with those with normal BMI, depending on the definition of multimorbidity used.

**Table 2.** The risk of multimorbidity in a 23-year follow-up by baseline BMI, waist circumference and body-roundness index using Cox regression analysis adjusting for baseline age and smoking status.

​	Chronic diseases over a 23-year follow-up (*n*)		
	Two or more	Three or more	Four or more
	aHR (95% CI)	aHR (95% CI)	aHR (95% CI)
**Women**			
**Body mass index (kg/m** ^**2**^ **) at baseline (*n* = 495)**			
18.5-24.9	ref.	ref.	ref.
25-29.9	1.29 (1.04-1.60)	1.42 (1.12-1.80)	1.39 (1.06-1.82)
≥30	1.75 (1.35-2.27)	2.04 (1.54-2.71)	2.17 (1.59-2.97)
**Waist circumference (cm) at baseline (*n* = 491)**			
<80	ref.	ref.	ref.
80-87.9	1.20 (0.95-1.49)	1.39 (1.05-1.84)	1.19 (0.86-1.64)
≥88	1.52 (1.21-1.92)	1.85 (1.44-2.38)	1.78 (1.34-2.36)
**Body roundness index at baseline (*n*= 491)**			
Lowest tertile (<3.13)	ref.	ref.	ref.
Intermediate tertile (3.13-4.43)	1.18 (0.92-1.50)	1.35 (1.03-1.77)	1.22 (0.90-1.67)
Highest tertile (>4.43)	1.56 (1.22-2.00)	1.77 (1.35-2.33)	1.74 (1.28-2.37)
**Men**			
**Body mass index (kg/m** ^**2**^ **) at baseline (*n* = 492)**			
<25	ref.	ref.	ref.
25-29.9	1.13 (0.92-1.40)	1.19 (0.94-1.51)	1.28 (0.98-1.67)
≥30	2.41 (1.82-3.19)	2.69 (2.00-3.63)	2.79 (2.00-3.90)
**Waist circumference (cm) at baseline (*n* = 491)**			
<94	ref.	ref.	ref.
94-101.9	1.25 (0.97-1.62)	1.25 (0.97-1.61)	1.16 (0.87-1.54)
≥102	2.45 (1.90-3.15)	2.42 (1.88-3.11)	2.54 (1.93-3.34)
**Body roundness index at baseline (*n* = 491)**			
Lowest tertile (<3.59)	ref.	ref.	ref.
Intermediate tertile (3.58-4.47)	1.19 (0.94-1.51)	1.09 (0.84-1.43)	1.12 (0.82-1.52)
Highest tertile (>4.47)	2.05 (1.62-2.60)	2.10 (1.62-2.71)	2.22 (1.67-2.96)

aHR: adjusted hazard ratio, CI: confidence interval, ref.: reference

Similarly, participants with a baseline WC above the obesity threshold (88 cm for women and 102 cm for men) had an increased risk of multimorbidity compared with those with normal WC (<80 cm for women and <94 cm for men), with hazard ratios of 1.52–1.78 (95 % CI: 1.21–2.38) for women and 2.45–2.54 (95 % CI: 1.88–3.34) for men. High baseline BRI was also a significant risk factor for multimorbidity. Being in the highest BRI tertile was associated with hazard ratios of 1.56–1.77 (95% CI: 1.22–2.37) in women and 2.05–2.22 (95% CI: 1.62–2.96) in men, compared with those in the lowest BRI tertile (Table 2). Hazard ratios for the adjustment variables (age and smoking status) are presented in Supplementary Table 2.

**Figure 2.**
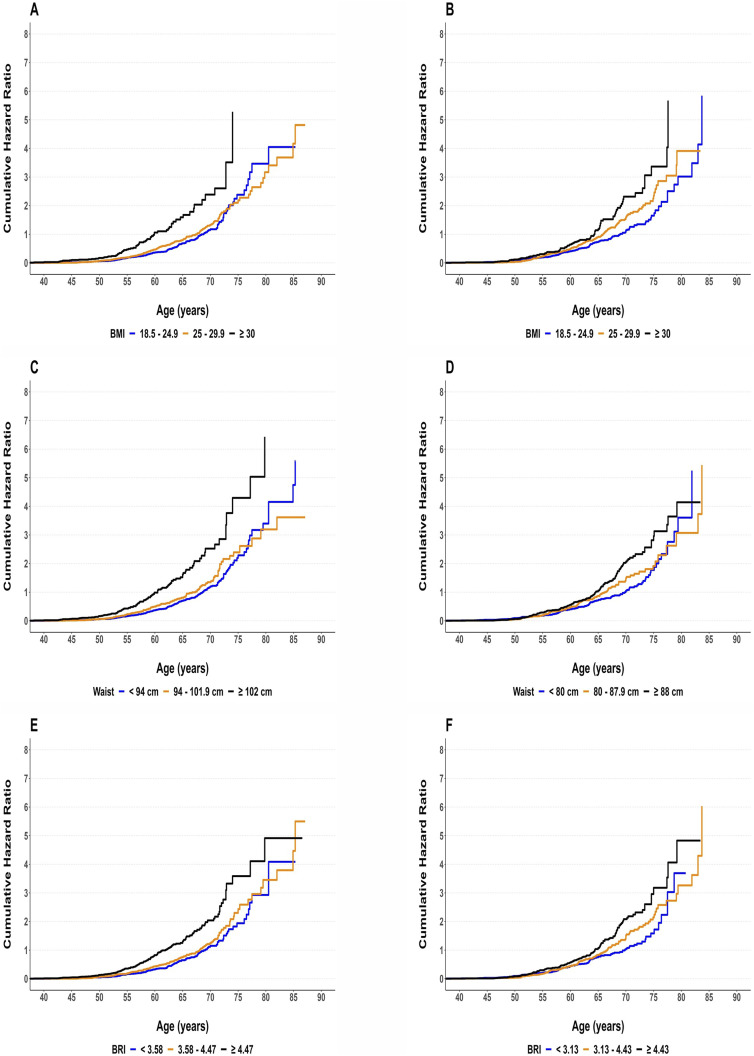
Cumulative hazard ratios for multimorbidity by baseline waist circumference, body mass index (BMI), and body roundness index (BRI) categories. Figures 2A, 2C, and 2E show data for men and 2B, 2D and 2F for women. Models are adjusted for study baseline age and smoking.

Figure 2(a)–(f) shows the cumulative hazard ratios for multimorbidity across BMI, WC, and BRI categories, presented separately for men and women. Alongside the standard definition of multimorbidity (≥2 chronic diseases), hazard ratio estimates based on additional definitions of multimorbidity (three or more and four or more chronic diseases) are provided in Supplementary Figures 2A–2F and 3A–3F.

**Figure 3.**
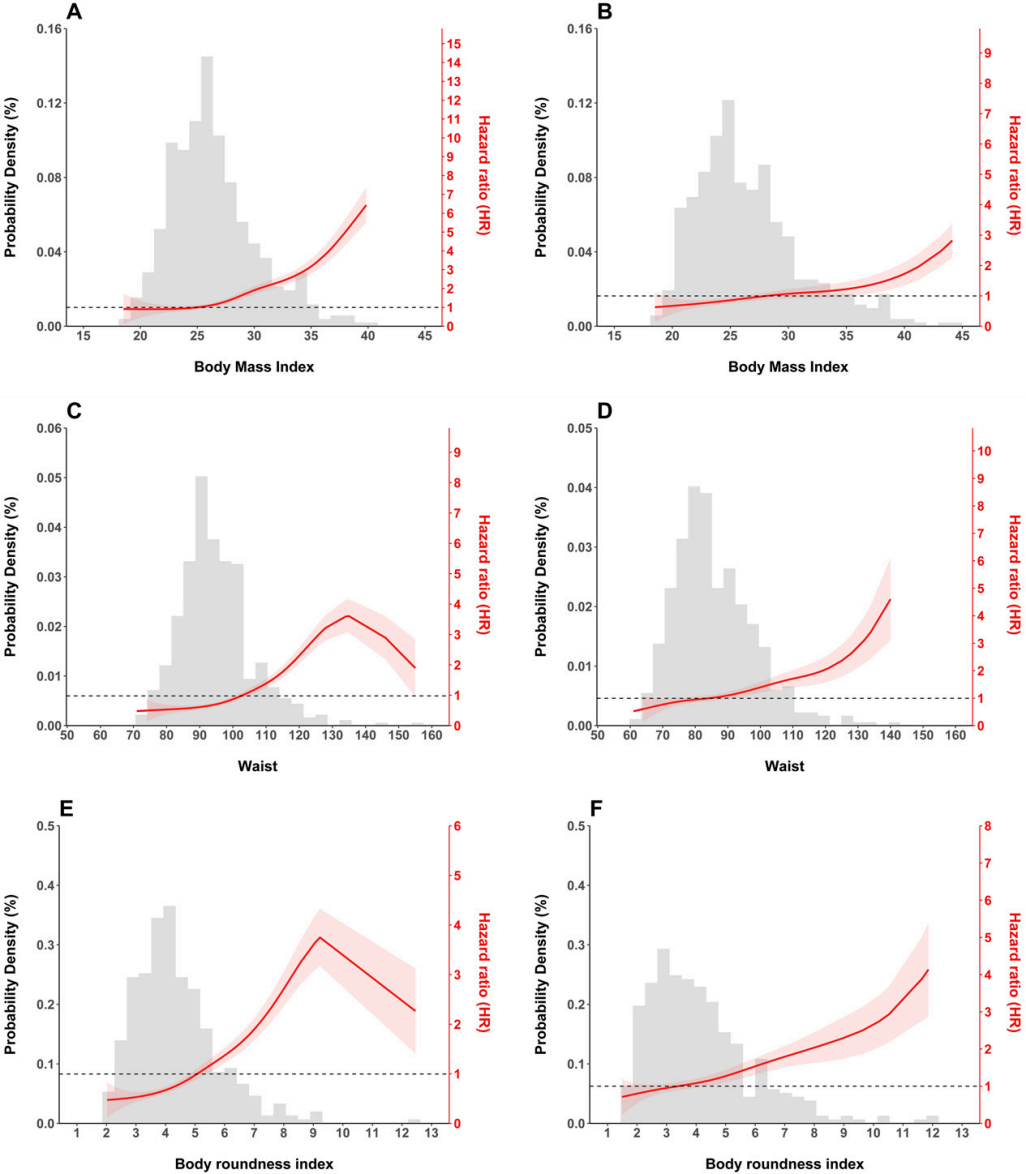
Associations between baseline body roundness index (BRI), body mass index (BMI) and waist circumference (WC) and the risk for multimorbidity over 23-year follow-up. Figures 3A, 3C, and 3E present associations for men, and 3B, 3D, and 3F for women. The solid curve illustrates the hazard ratio for multimorbidity, and the shading indicating the 95% CI. Bars represent the probability densities of BRI, BMI (kg/m2), and WC (cm), indicating the relative distribution of these anthropometric values in the study population.

Figure 3(a)–(f) illustrates the role of baseline BMI, WC, and BRI as risk factors for multimorbidity over a 23-year follow-up, with participants remaining free of multimorbidity (0–1 chronic disease) throughout follow-up serving as the reference group. The number of participants with the highest WC and BRI values was low, which affected the statistical analyses and broadened the 95% confidence intervals.

In sensitivity analyses assessing potential confounding, obesity defined by BMI remained a statistically significant risk factor for later multimorbidity compared with normal weight participants for both men and women in analyses adjusted for SES and, separately, for educational level. Obesity defined by WC also remained significantly associated with multimorbidity in analyses adjusted for SES and educational level among men (aHR 3.52, 95% CI 2.20–5.64 and aHR 2.80, 95 % CI 1.82–4.31, respectively). Among women, however, the association was attenuated and no longer statistically significant in analyses adjusted for SES (aHR 1.21, 95% CI 0.83–1.78) or educational level (aHR 1.28, 95% CI 0.91–1.80). The results of the sensitivity analyses, including those for complex multimorbidity (≥3 and ≥4 diseases), are presented in Supplementary Tables 3–5. When included in the models as the sole explanatory variable, no statistically significant associations with multimorbidity were observed across SES or educational levels.

ROC analysis identified cut-off points in baseline BMI, WC, and BRI that best predicted future multimorbidity, optimising the sensitivity and specificity (Supplementary table 6). The sex-specific thresholds were 26.4 (women) and 26.1 (men) for BMI, 76.3 cm and 93.3 cm for WC, and 3.41 and 3.70 for BRI, respectively.

## Discussion

### Main results

This 23-year prospective cohort study provides further evidence that midlife obesity – whether assessed using BMI, WC, or BRI – is a risk factor for multimorbidity with advancing age. The effects of elevated BMI and large WC on multimorbidity risk are more pronounced in men than in women.

### Comparison to existing literature

The present findings are consistent with previous studies demonstrating that obesity is a risk factor for multiple chronic diseases and their co-occurrence, i.e. multimorbidity.^44^ A recent meta-analysis of longitudinal studies reported a pooled risk ratio of 1.99 (95 % CI 1.45–2.72) for multimorbidity among individuals with BMI-defined obesity compared with those with normal weight.^19^ However, the available meta-analytic evidence largely reflects overall associations, as sex-specific risk estimates have been inconsistently reported in previous studies; notably, the authors also highlighted the importance of reporting sex-specific associations in future research.^19^ Evidence from longitudinal cohort studies on sex-specific obesity-related risks remain limited, although a previous analysis suggests sex-specific disease profiles, with stronger links to diabetes-related comorbidity in men and hypertension-related comorbidity in women.^45^

Compared with much of the existing literature, which has predominantly relied on BMI as the primary measure of adiposity, our study extends current evidence by demonstrating consistent associations across multiple adiposity measures, including waist circumference and body roundness index. In addition, our findings show that midlife obesity remains a risk factor for later multimorbidity when higher disease thresholds are applied, in both men and women.

To our knowledge, multimorbidity with a wide range of diagnoses has not been previously described in such detail, using such comprehensive methods in a Northern European population over a follow-up period exceeding 20 years.

Both obesity defined by BMI and WC, as well as high BRI, were associated with an increased risk of multimorbidity in both men and women. Although BMI and WC cut-off points are clinically useful for defining obesity,^46^ the risk of multimorbidity was already elevated at lower values.

This study also provides the first sex-specific BRI cut-offs for multimorbidity prediction, identifying thresholds of 3.41 for women and 3.70 for men. Although higher BRI values were significantly associated with an increased risk of multimorbidity, the modest AUC of 0.67 for these ROC-derived thresholds indicates limited discriminatory performance for a single cut-off value. Similar modest AUC values were observed for BMI and WC, suggesting that anthropometric measures are better interpreted as indicators of progressively increasing risk rather than precise tools for categorizing individuals based on fixed thresholds.

### Strengths and weaknesses

The study population consisted of a population-based sample of adults residing in a single Finnish municipality followed over a 23-year period. The study cohort is representative of the adult population of the municipality and could also be considered broadly representative of the wider region. The availability of comprehensive medical records and clinical examinations has provided a reliable basis for studying the development of multimorbidity in late adulthood.

The proportion of deaths is biased in the visualisation of the Sankey diagram in the older ages, as the diagram only accounts for those individuals who died during the follow-up period. Given that many individuals did not reach the age of 80 during the follow-up, the survival rates shown in the diagram are not representative.

Comprehensive data from healthcare registers were used to define chronic diseases in the study population. Self-reported diagnoses often underestimate the actual prevalence of chronic diseases.^47^ In this study, all diagnoses were physician-defined and documented. A total of 38 diagnoses were used to define multimorbidity, which was categorized as ≥2, ≥3, and ≥4 diagnoses. Using more than 12 diagnoses and three different operational definitions of multimorbidity align with the suggestions of Fortin et al.^22^ Objectively measured values of BMI and WC were used to define overweight and obesity avoiding potential biases of self-reported values, commonly used in other reports. In addition, we evaluated the value of BRI as the risk factor for multimorbidity.

Alternative approaches to disease counts have been developed for predicting different outcomes of interest.^48^ The simple count of diseases has been shown to have a good predictive value for many endpoints – performing almost as effectively as more complex measures, especially for care utilisation and quality of life.^49^

Nevertheless, some diagnoses, particularly those in mild or moderate stages, may be missing, if they were not recorded in medical records. In contrast, the documented diagnoses are likely to be clinically relevant. The number of dropouts and deaths was considerable during the follow-up, mainly due to the age range of the population. Diagnoses recorded during clinic or hospital visits, as well as those inferred from prescribed drugs, were included, however information from death certificates was not considered.

The severity and impact of these diseases on daily activities cannot be determined from the available data. Some diseases may have been cured through medication or surgical interventions. However, a cured disease does not necessarily imply full recovery of function or quality of life. For example, curing cancer may involve multiple medications and potential adverse effects from treatments. The diagnoses selected represent common, care-requiring chronic conditions in this population and reflect the associated burden on health-care services, consistent with recommended principles for defining multimorbidity.^36,38^

The list of diseases in the present study differs from the recent Delphi consensus study,^38^ which defines and recommends always including 24 specific diseases. The present study included 38 diagnoses but did not include the recommended Addison’s disease, AIDS/HIV infection, or cystic fibrosis, as these conditions occurred in fewer than five participants in the study population and are very rare in the population. These diagnoses were therefore omitted and excluded prior to conducting the analyses. The consensus study recommendation^38^ also highlights the need to adapt the list of diseases to ensure its relevance to the study population, and the exclusion of rare diagnoses has also been adopted in previous studies.^50,51^

Changes in BMI, BRI, and WC during follow-up were not analysed in the present study. A total of 349 women and 270 men from the original Savitaipale Study population participated in the 22-year follow-up examination. According to previously published analyses based on these participants, BMI remained statistically unchanged in both men and women. In contrast, mean waist circumference increased significantly from 85.6 cm to 90.6 cm (p < 0.001) in women and from 94.6 cm to 99.0 cm (p < 0.001) in men.^32^ Based on this subset of participants with follow-up measurements, the observed increase in waist circumference over time may have contributed to the risk of multimorbidity.

A key limitation of this study is that information on SES and educational level was not available at baseline for the study population. These data were collected more comprehensively only during later follow-up examinations, which not all participants attended. Consequently, SES and education could not be included as baseline covariates in the main analyses. To address this, sensitivity analyses were conducted among participants with available information on SES and education.

While obesity, defined by BMI, remained a robust risk factor for multimorbidity after adjusting for SES and educational level in both men and women, the association with WC appeared to be attenuated among women in sensitivity analyses. This attenuation was observed only in analyses conducted in a restricted subgroup and should therefore be interpreted with caution, as it may reflect limited statistical power or potential selection bias rather than a true absence of association.

A further limitation relates to individuals with underweight. As only five individuals were classified as underweight according to BMI, this category was excluded from the study population due to concerns regarding participant confidentiality. Therefore, individuals with underweight were not represented in the present analyses, and the findings may not be generalizable to this population.

In addition, other lifestyle-related factors, such as physical activity, dietary habits, and sleep, which may potentially influence the risk of multimorbidity,^52–55^ were not accounted for in the analyses and may have contributed to residual confounding.

Obesity is thought to contribute to the development of multimorbidity through multiple interrelated biological mechanisms. Excess adiposity is associated with chronic low-grade systemic inflammation, insulin resistance, and dysregulated lipid metabolism, all of which play a central role in the pathogenesis of cardiometabolic diseases. ^^56,57^^ Adipose tissue also functions as an active endocrine organ, secreting adipokines and pro-inflammatory cytokines that may promote endothelial dysfunction, atherosclerosis, and metabolic dysregulation.^^58^^ In addition, obesity has been linked to fibrogenesis and ectopic fat deposition in organs such as the liver, pancreas, and skeletal muscle, potentially contributing to impaired organ function and beta-cell dysfunction.^^58^^ Beyond metabolic pathways, excess body weight increases mechanical load on joints and other tissues, thereby elevating the risk of musculoskeletal disorders such as osteoarthritis.^^59^^ Together, these metabolic, inflammatory, and mechanical pathways may partly explain why obesity predisposes individuals to the accumulation of multiple chronic conditions over time.^^58,60^^

Diagnostic practices and coding standards evolve over time, and ICD-10 is more granular than ICD-9. To ensure comparability, we manually reviewed ICD-10 codes and identified clinically equivalent ICD-9 codes, cross-checking the mapping against a prior publication.^10^ Although differences between coding systems are most pronounced in detailed subcategories,^^61^^ in this study we focused on common long-term conditions, for which Finnish health-care registers have demonstrated good validity.^^33^^ To confirm completeness, we also manually examined diagnoses that initially fell outside the predefined extraction codes and used these to supplement the final ICD-9/ICD-10 code list presented in Supplementary Table 1.

## Conclusions

These findings from long-term follow-up add longitudinal evidence that obesity in midlife is associated with the development of multimorbidity in later life. Despite long-standing recognition of its adverse health effects, obesity prevalence is projected to increase, highlighting the need for effective lifelong prevention and management at both societal and health care system levels.

## Supplemental material

Supplemental material - Obesity as a risk factor for multimorbidity: 23-Years population-based prospective study Supplemental material for Obesity as a risk factor for multimorbidity: 23-Years population-based prospective study by Jussa-Pekka Välitalo, Paula Pesonen, Jouko Saramies, Sirkka Keinänen-Kiukaanniemi, Hannu Uusitalo, Julia Lybeck, Esko Hussi, Kadri Suija, Jaakko Tuomilehto and Juha Auvinen in Journal of Multimorbidity and Comorbidity.

## Data Availability

The data that support the findings of this study are not publicly available due to privacy regulations.*

## References

[1] Van Den Akker M Buntinx F Knottnerus JA . Comorbidity or multimorbidity. Eur J Gen Pract 1996; 2(2): 65–70. 10.3109/13814789609162146

[2] Boyd CM Fortin M . Future of Multimorbidity Research: How Should Understanding of Multimorbidity Inform Health System Design. Public Health Reviews 2010; 32(2): 451–474. doi:10.1007/BF03391611

[3] Valderas JM Starfield B Sibbald B , et al.. Defining comorbidity: implications for understanding health and health services. Ann Fam Med 2009; 7(4): 357–363. 10.1370/AFM.983 19597174 PMC2713155

[4] Farmer C Fenu E O’Flynn N , et al.. Clinical assessment and management of multimorbidity: Summary of NICE guidance. BMJ (Online) 2016; 354: i4843. 10.1136/bmj.i4843 27655884

[5] Diane Zheng D Loewenstein DA Christ SL , et al. Multimorbidity patterns and their relationship to mortality in the US older adult population. PLoS One 2021; 16(1): e0245053. 10.1371/JOURNAL.PONE.0245053 33471812 PMC7816983

[6] Wikström K Lindström J Harald K , et al.. Clinical and lifestyle-related risk factors for incident multimorbidity: 10-year follow-up of Finnish population-based cohorts 1982-2012. Eur J Intern Med 2015; 26(3): 211–216. 10.1016/j.ejim.2015.02.012 25747490

[7] Soley-Bori M Ashworth M Bisquera A , et al. Impact of multimorbidity on healthcare costs and utilisation: a systematic review of the UK literature. British Journal of General Practice 2021; 71(702): e39–e46. 10.3399/bjgp20X713897 PMC7716874 33257463

[8] Triolo F Harber-Aschan L Belvederi MM , et al. The complex interplay between depression and multimorbidity in late life: risks and pathways. Mech Ageing Dev 2020; 192: 111383. 10.1016/j.mad.2020.111383 33045250

[9] Kanesarajah J Waller M Whitty JA , et al.. Multimorbidity and quality of life at mid-life: A systematic review of general population studies. Maturitas 2018; 109: 53–62. 10.1016/j.maturitas.2017.12.004 29452782

[10] Junttila O Pesonen P Timonen M , et al.. Multimorbidity and health related quality of life in midlife–a longitudinal study from Northern Finland Birth Cohort 1966. Scand J Prim Health Care 2025. Published online. 10.1080/02813432.2025.2492296;WGROUP:STRING:PUBLICATION PMC12918400 40285329

[11] Barnett K Mercer SW Norbury M , et al.. Epidemiology of multimorbidity and implications for health care, research, and medical education: A cross-sectional study. The Lancet 2012; 380(9836): 37–43. 10.1016/S0140-6736(12)60240-2 22579043

[12] Chowdhury SR Chandra Das D Sunna TC , et al.. Global and regional prevalence of multimorbidity in the adult population in community settings: a systematic review and meta-analysis. EClinicalMedicine 2023; 57: 101860. 10.1016/j.eclinm.2023.101860 36864977 PMC9971315

[13] Obesity and overweight. Accessed October 26, 2024. https://www.who.int/news-room/fact-sheets/detail/obesity-and-overweight

[14] Janssen F Bardoutsos A Vidra N . Obesity Prevalence in the Long-Term Future in 18 European Countries and in the USA. Obes Facts 2020; 13: 514–527. 10.1159/000511023 33075798 PMC7670332

[15] Booth HP Prevost AT Gulliford MC . Impact of body mass index on prevalence of multimorbidity in primary care: cohort study. Fam Pract 2014; 31(1): 38–43. 10.1093/FAMPRA/CMT061 24132593 PMC3902211

[16] Kivimäki M Kuosma E Ferrie JE , et al. Overweight, obesity, and risk of cardiometabolic multimorbidity: pooled analysis of individual-level data for 120 813 adults from 16 cohort studies from the USA and Europe. Lancet Public Health 2017; 2(6): e277–e285. 10.1016/S2468-2667(17)30074-9 28626830 PMC5463032

[17] Andersén H Kankaanranta H Tuomisto LE , et al. Multimorbidity in Finnish and Swedish speaking Finns; association with daily habits and socioeconomic status – Nordic EpiLung cross-sectional study. Prev Med Rep 2021; 22: 101338. 10.1016/j.pmedr.2021.101338 33732608 PMC7937573

[18] Bezerra de Souza DL Oliveras-Fabregas A Espelt A , et al. Multimorbidity and its associated factors among adults aged 50 and over: A cross-sectional study in 17 European countries. PLoS One 2021; 16(2): e0246623. 10.1371/JOURNAL.PONE.0246623 33571285 PMC7877625

[19] Delpino FM dos Santos Rodrigues AP Petarli GB , et al. Overweight, obesity and risk of multimorbidity: A systematic review and meta-analysis of longitudinal studies. Obesity Reviews 2023; 24(6): e13562. 10.1111/obr.13562 36929143

[20] Blümel JE Carrillo-Larco RM Vallejo MS , et al.. Multimorbidity in a cohort of middle-aged women: Risk factors and disease clustering. Maturitas 2020; 137: 45–49. 10.1016/J.MATURITAS.2020.04.016 32498936 PMC7284304

[21] Xu X Mishra GD Dobson AJ , et al.. Short-term weight gain is associated with accumulation of multimorbidity in mid-aged women: a 20-year cohort study. Int J Obes (Lond) 2019; 43(9): 1811–1821. 10.1038/S41366-018-0250-7 30459401

[22] Fortin M Stewart M Poitras ME , et al.. A Systematic Review of Prevalence Studies on Multimorbidity: Toward a More Uniform Methodology. Ann Fam Med 2012; 10(2): 142–151. 10.1370/AFM.1337 22412006 PMC3315131

[23] Bramhankar M Pandey M Rana GS , et al.. An assessment of anthropometric indices and its association with NCDs among the older adults of India: evidence from LASI Wave-1. BMC Public Health 2021; 21(1): 1357. 10.1186/S12889-021-11421-4 34238276 PMC8268209

[24] Olivares DEV Chambi FRV Chañi EMM , et al. Risk Factors for Chronic Diseases and Multimorbidity in a Primary Care Context of Central Argentina: A Web-Based Interactive and Cross-Sectional Study. International Journal of Environmental Research and Public Health 2017; 14(3): 251. 10.3390/IJERPH14030251 28257087 PMC5369087

[25] Ye X Shi D Li R , et al. Association between weight-adjusted waist index (WWI) and multimorbidity: Evidence from NHANES and prospective validation in CHARLS. Exp Gerontol 2026; 215: 113044. 10.1016/J.EXGER.2026.113044 41577092

[26] Thomas DM Bredlau C Bosy-Westphal A , et al. Relationships between body roundness with body fat and visceral adipose tissue emerging from a new geometrical model. Obesity (Silver Spring) 2013; 21(11): 2264–2271. 10.1002/OBY.20408 23519954 PMC3692604

[27] Geng S Chen X Shi Z , et al.. Association of anthropometric indices with the development of multimorbidity in middle-aged and older adults: A retrospective cohort study. PLoS One 2022; 17(10): e0276216. 10.1371/journal.pone.0276216 36240163 PMC9565419

[28] Kunutsor SK Bhattacharjee A Jae SY , et al.. Body roundness index and cardiometabolic multimorbidity: Findings from the English Longitudinal Study of Ageing. Nutrition, Metabolism and Cardiovascular Diseases 2026; 36(4): 104475. 10.1016/j.numecd.2025.104475 41478746

[29] Sun Y Sun P Liu J , et al.. The Association Between Body Roundness Index and Cardiovascular Disease Risk: An Analysis Based on the CHARLS Database. Rev Cardiovasc Med 2025; 26(9): 39048. 10.31083/RCM39048 41089772 PMC12516779

[30] World health organization . Obesity: preventing and managing the global epidemic. Report of a WHO Consultation on Obesity. Geneva, 1997 (WHO/NUT/NCD/98.1). World Health Organ Tech Rep Ser 2000;894:(1): 1-8. 11234459

[31] Abbafati C Abbas KM Abbasi-Kangevari M , et al. Global burden of 87 risk factors in 204 countries and territories, 1990–2019: a systematic analysis for the Global Burden of Disease Study 2019. The Lancet 2020; 396(10258): 1223–1249. 10.1016/S0140-6736(20)30752-2 PMC7566194 33069327

[32] Saramies J Koiranen M Auvinen J , et al. 22-year trends in dysglycemia and body mass index: A population-based cohort study in Savitaipale, Finland. Prim Care Diabetes 2021; 15(6): 977–984. 10.1016/J.PCD.2021.09.010 34649826

[33] Sund R . Quality of the Finnish Hospital Discharge Register: a systematic review. Scand J Public Health 2012; 40(6): 505–515. 10.1177/1403494812456637 22899561

[34] Laugesen K Ludvigsson JF Schmidt M , et al. Nordic Health Registry-Based Research: A Review of Health Care Systems and Key Registries. Clin Epidemiol 2021; 13: 533–554. 10.2147/CLEP.S314959 34321928 PMC8302231

[35] Griffith LE Gruneir A Fisher KA , et al. Key factors to consider when measuring multimorbidity: Results from an expert panel and online survey. J Comorb 2018; 8(1): 2235042X1879530. 10.1177/2235042X18795306 PMC6169974 30363320

[36] Diederichs C Berger K Bartels DB . The Measurement of Multiple Chronic Diseases—A Systematic Review on Existing Multimorbidity Indices. The Journals of Gerontology: Series A 2011; 66A(3): 301–311. 10.1093/GERONA/GLQ208 21112963

[37] Calderón-Larrañaga A Vetrano DL Onder G , et al. Assessing and Measuring Chronic Multimorbidity in the Older Population: A Proposal for Its Operationalization. Journals of Gerontology - Series A Biological Sciences and Medical Sciences 2017; 72(10): 1417–1423. 10.1093/GERONA/GLW233 28003375 PMC5861938

[38] Ho ISS Azcoaga-Lorenzo A Akbari A , et al. Measuring multimorbidity in research: Delphi consensus study. BMJ Medicine 2022; 1(1): e000247. 10.1136/BMJMED-2022-000247 36936594 PMC9978673

[39] Alberti KGMM Zimmet P Shaw J . Metabolic syndrome—a new world-wide definition. A Consensus Statement from the International Diabetes Federation. Diabetic Medicine 2006; 23(5): 469–480. 10.1111/J.1464-5491.2006.01858.X 16681555

[40] Dai X Gil GF Reitsma MB , et al. Health effects associated with smoking: a Burden of Proof study. Nat Med 2022; 28(10): 2045–2055. 10.1038/S41591-022-01978-X 36216941 PMC9556318

[41] Leffondré K Abrahamowicz M Siemiatycki J , et al.. Modeling smoking history: a comparison of different approaches. Am J Epidemiol 2002; 156(9): 813–823. 10.1093/aje/kwf122 12396999

[42] Pathirana TI Jackson CA . Socioeconomic status and multimorbidity: a systematic review and meta-analysis. Aust N Z J Public Health 2018; 42(2): 186–194. 10.1111/1753-6405.12762 29442409

[43] Hanley JA McNeil BJ . A method of comparing the areas under receiver operating characteristic curves derived from the same cases. Radiology 1983; 148(3): 839–843. 10.1148/radiology.148.3.6878708 6878708

[44] Haslam DW James WPT . Obesity. Lancet 2005; 366(9492): 1197–1209. 10.1016/S0140-6736(05)67483-1 16198769

[45] Ma L Li Y Li G , et al. Adiposity indicators exhibit depot- and sex-specific associations with multimorbidity onset: A cohort study of the UK Biobank. Diabetes Obes Metab 2024; 26(7): 2890–2904. 10.1111/dom.15610 38686512

[46] Zhang L Liu Z Si D , et al. Comparative predictive values of anthropometric indices for cardiometabolic multimorbidity in middle-aged and older adults: a prospective study from the CHARLS study. BMC Public Health 2025; 26: 333. 10.1186/S12889-025-26060-2 41437013 PMC12838432

[47] Liu H Zhao Y Qiao L , et al. Consistency between self-reported disease diagnosis and clinical assessment and under-reporting for chronic conditions: data from a community-based study in Xi’an, China. Front Public Health 2024; 12: 1296939. 10.3389/FPUBH.2024.1296939/BIBTEX 38292908 PMC10825002

[48] Johnston MC Crilly M Black C , et al.. Defining and measuring multimorbidity: a systematic review of systematic reviews. Eur J Public Health 2019; 29(1): 182–189. 10.1093/EURPUB/CKY098 29878097

[49] Huntley AL Johnson R Purdy S , et al.. Measures of multimorbidity and morbidity burden for use in primary care and community settings: A systematic review and guide. Ann Fam Med 2012; 10(2): 134–141. 10.1370/AFM.1363 22412005 PMC3315139

[50] Marques LP de Aguiar OB Paula DP , et al. Multimorbidity prevalence and patterns at the baseline of the Brazilian Longitudinal Study of Adult Health (ELSA-Brasil). Journal of Multimorbidity and Comorbidity 2023; 13: 26335565231173845. 10.1177/26335565231173845 37223823 PMC10201182

[51] Heikkala E Mikkola I Jokelainen J , et al.. Multimorbidity and achievement of treatment goals among patients with type 2 diabetes: a primary care, real-world study. BMC Health Serv Res 2021; 21(1): 964. 10.1186/s12913-021-06989-x 34521389 PMC8442281

[52] Shao J Wang X Zou P , et al. Associating modifiable lifestyle factors with multimorbidity in community dwelling individuals from mainland China. European Journal of Cardiovascular Nursing 2021; 20(6): 556–564. 10.1093/EURJCN/ZVAA038 33580782

[53] Keramat SA Alam K Basri R , et al. Sleep quality, obesity and the risk of multimorbidity among Australian middle-aged and older adults: Evidence from a national longitudinal household survey. Aust N Z J Public Health 2025; 49(6): 100280. 10.1016/J.ANZJPH.2025.100280 41162264

[54] Dhalwani NN Zaccardi F O’Donovan G , et al. Association Between Lifestyle Factors and the Incidence of Multimorbidity in an Older English Population. J Gerontol A Biol Sci Med Sci 2017; 72(4): 528–534. 10.1093/GERONA/GLW146 27470302

[55] Zhang Y Chen H Carrillo-Larco RM , et al. Association of dietary patterns and food groups intake with multimorbidity: A prospective cohort study. Clin Nutr ESPEN 2022; 51: 359–366. 10.1016/j.clnesp.2022.07.019 36184228

[56] Tchkonia T Thomou T Zhu Yi Karagiannides I Pothoulakis C , et al. Mechanisms and Metabolic Implications of Regional Differences among Fat Depots. Cell Metabolism 2013; 17(5): 644-656. https://linkinghub.elsevier.com/retrieve/pii/S1550413113001113 23583168 10.1016/j.cmet.2013.03.008 PMC3942783

[57] Kawai T Autieri MV Scalia R . Adipose tissue inflammation and metabolic dysfunction in obesity. American Journal of Physiology-Cell Physiology 2021; 320(3): C375-C391. 10.1152/ajpcell.00379.2020 https://journals.physiology.org/doi/10.1152/ajpcell.00379.2020 33356944 PMC8294624

[58] Jin X Qiu T Li L Yu R Chen X , et al. Pathophysiology of obesity and its associated diseases. Acta Pharmaceutica Sinica B 2023; 13(6): 2403-2424. https://linkinghub.elsevier.com/retrieve/pii/S2211383523000126 37425065 10.1016/j.apsb.2023.01.012 PMC10326265

[59] Goldring MB Otero M . Inflammation in osteoarthritis. Current Opinion in Rheumatology 2011; 23(5): 471-478. https://journals.lww.com/00002281-201109000-00012 21788902 10.1097/BOR.0b013e328349c2b1 PMC3937875

[60] Heymsfield SB Wadden TA . Mechanisms, Pathophysiology, and Management of Obesity. Longo DL New England Journal of Medicine 2017; 376(3): 254-266. http://www.nejm.org/doi/10.1056/NEJMra1514009 28099824 10.1056/NEJMra1514009

[61] Cartwright DJ . ICD-9-CM to ICD-10-CM Codes: What? Why? How?. Advances in Wound Care 2013; 2(10): 588-592. https://journals.sagepub.com/doi/full/10.1089/wound.2013.0478 24761333 10.1089/wound.2013.0478 PMC3865615

