# Supplementary material for: Obesity as a risk factor for multimorbidity: a 23-years population-based prospective study
Source: J Multimorb Comorb. 2026 Aug 17;16:26335565261478101. doi: 10.1177/26335565261478101 (PMC13487119; doi:10.1177/26335565261478101)
Supplement: Supplementary file 1 [file sj-pdf-1-cob-10.1177_26335565261478101.pdf]

Supplementary file – Obesity as a risk factor for multimorbidity: 23-years population-based prospective study

Supplementary Table 1: Identification of 38 common chronic diseases based on national and local health care registers.

**Disease**	**Diagnosise codes (ICD-10) from national Register of Primary Health Care visits (years 2011-2019), Care Register for Health Care (1996-2019) and local primary care (1996-2010) and secondary health care records (2005-2019)**	**Diagnosise codes (ICD-9) from (years 1986-1995)**	**Use of prescribed drugs (ATC-code) from the national medication purchase register of the National Social Insurance Institution (years 1994-2019) and local heath register (2005-2019)**	**Codes for the reimbursement of drug costs for certain diseases from the National Social Insurance Institution register (years 1964-2019)**
Malignant neoplasms	C00-C97	140-199, 200-208		115, 116, 117, 128, 130
Anaemias	D50-58	280-285		122, 126, 129
Sarcoidosis	D86	135		132
Hypothyroidism	E03	243-244	H03A	104
Hyperthyroidism	E05	242	H03B	
Type 2 diabetes mellitus	E11	250A, 250C and 250X	A10B	215, 285
Schizophrenia and schizoaffective disorders	F20 and F25	295		
Mania and bipolar affective disorder	F30-F31, excluding F30.0	2962, 2963, 2964, 2967A		
Depression	F32-F33, F34.1	2961, 2968A		
Anxiety, dissociative and somatoform disorders	F40-42 and F44-F45	300		
Parkinson disease	G20	3320A	N04BA, N04BD, N04BX	110
Alzheimer disease and vascular dementia	G30, F00*, F01	3310A	N06DA, N06DX01	
Demyelinating diseases of the central nervous system	G35-G37	340, 341		109, 157, 303
Epilepsy	G40-G41	345		111, 181, 182, 183, 199,

Migraine	G43	346	N02CC, N02CD	
Transient ischaemic attac and strokes	G45 and I60-I63.9, I69.1-69.3	430-435	B01AC07	
Retinopathy	H35	362		
Cataract	H25-H26	366		
Glaucoma	H40.1-H40.9	365		114
Hypertension	I10	401-404		205
Ischemic heart diseases	I20-I25 and Z95.1 excluding 20.00, 20.10, 20.80, 20.90	410-414	C01DA	206
Atrial fibrillation or atrial flutter	I48	4273A		290, 342, 351
Heart failure	I50, I11.0, I13.0, I13.2	4029, 428	C09DX04	201, 381, 289
Peripheral arterial disease	I70.2	4402A, 4408X		
COPD	J44.1, J44.8, J44.9	4912A, 4912B, 496	R03BB, R03AL	
Asthma	J45-J46	493	R03DC03, R03BA	
Psoriasis	L40	6960A, 6961A, 6961B	D05A	319, 377
Inflammatory polyarthritis and systemic connective tissue disorders	M05-M14, M34-M36 and M45, excluding M10	710, 714, 7165A	M01CB	202, 313
Osteoarthritis	M15-M18.9 and M19.8	715		
Osteoporosis	M80-M81	7330A, 7330B	M05B	318
Endometriosis	N80	617		
Prostate hyperplasia	N40	600	G04CA	
Gout	M10	274	M04AA, M04AB	212
Kidney diseases	E10.2, E11.2, I12, I13, N03, N04#, N05#, N12, N08.3#, N18, N19 and Z49	581, 582, 585		
Inflammatory bowel diseases	K50-K52	555, 556	L01BB02, A07EC02, A07EA	208
Coeliac disease	K90.0	5790A		
Alcohol use disorder	F10	303	N07BB04	
Sleep apnoea	G47.3	327.23		

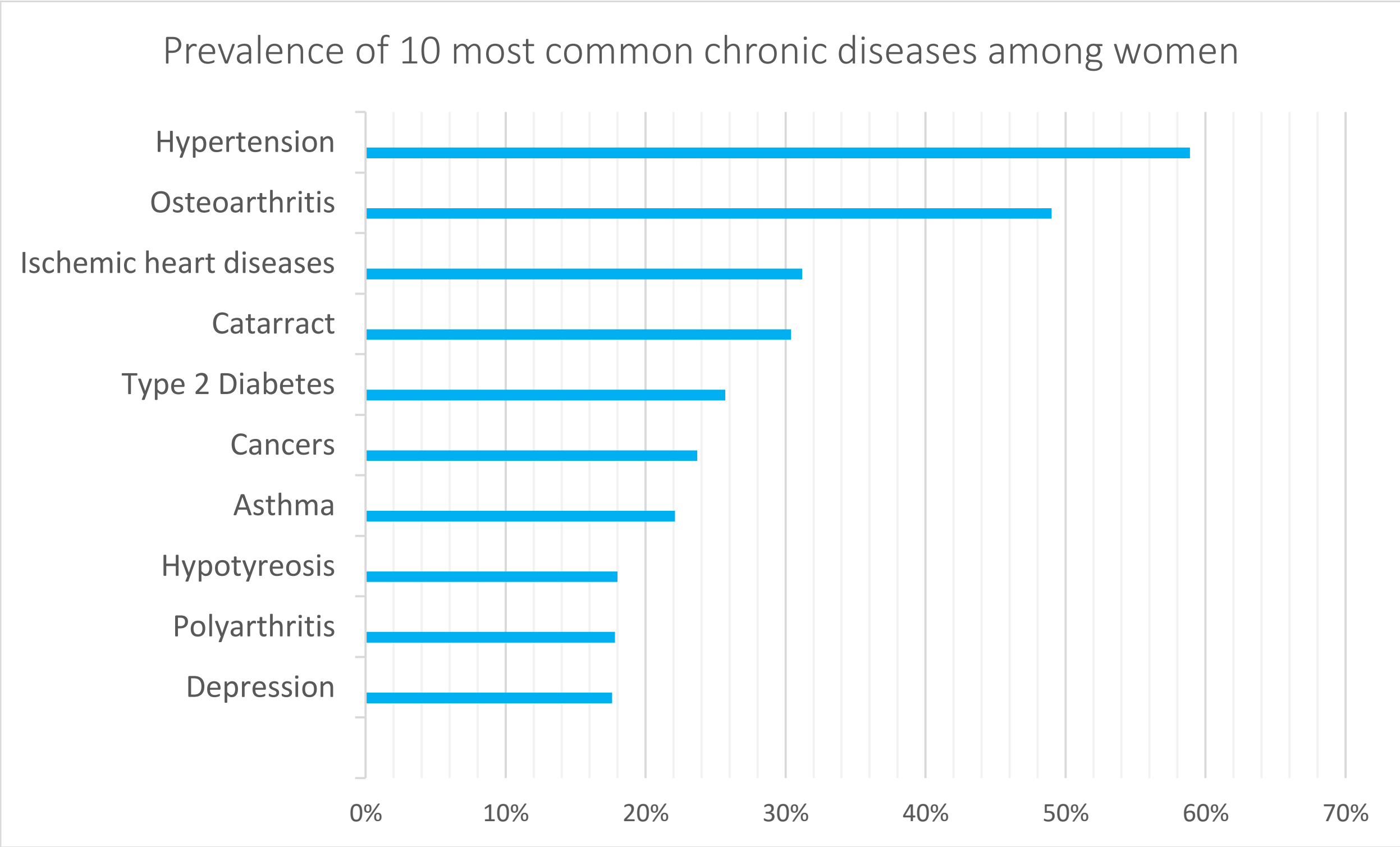

Supplementary Figure 1A. 10 most prevalent chronic diseases among women during the 23-year follow-up.

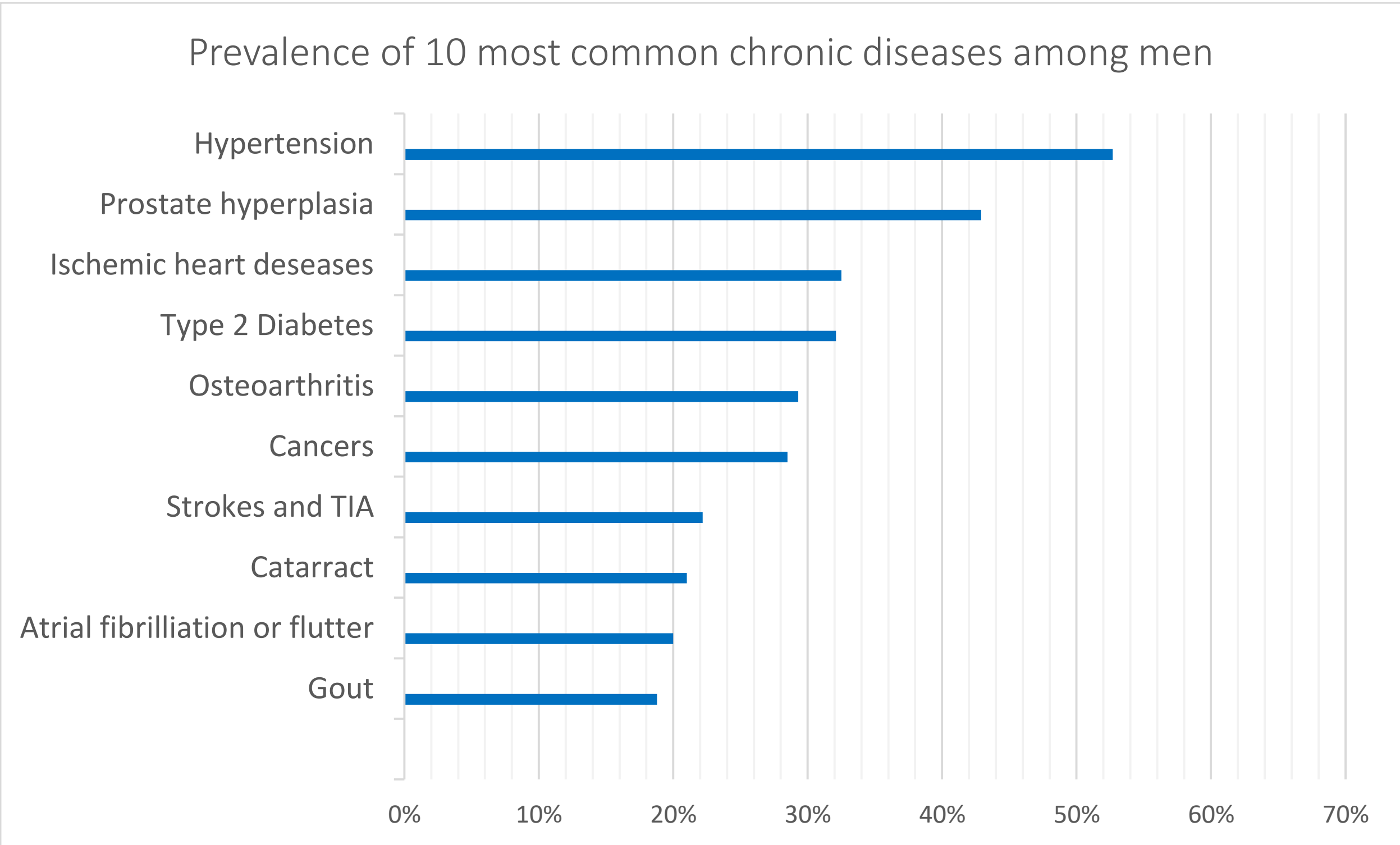

Supplementary Figure 1B. 10 most prevalent chronic diseases among men during the 23-year follow-up.

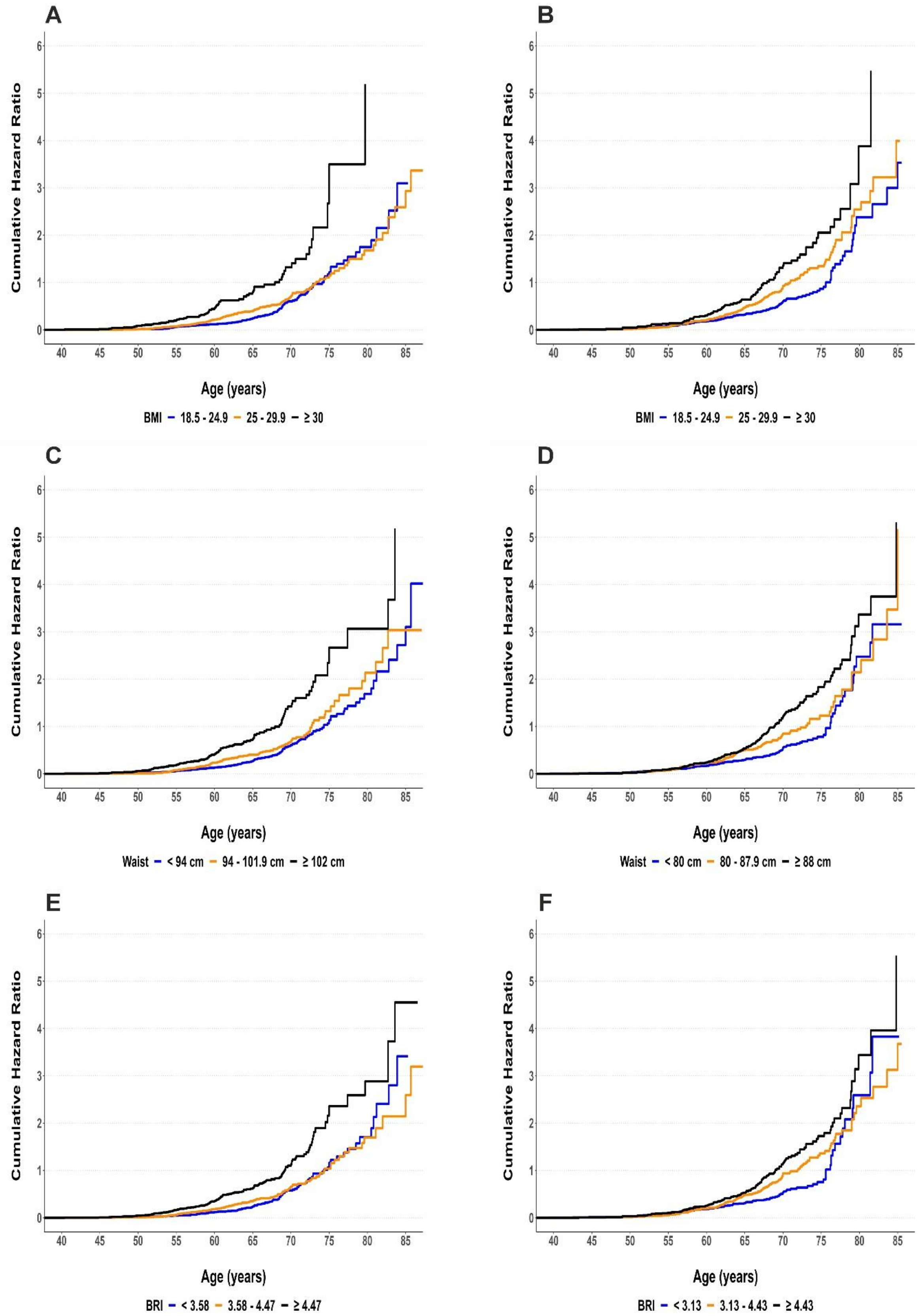

Supplementary figure 2A-2F. Cumulative hazard ratios for multimorbidity (≥3 diseases) by baseline waist circumference, body mass index (BMI), and body roundness index (BRI) categories. Supplementary Figures 2A, 2C, and 2E show data for men and 2B, 2D and 2F for women. Models are adjusted for study baseline age and smoking.

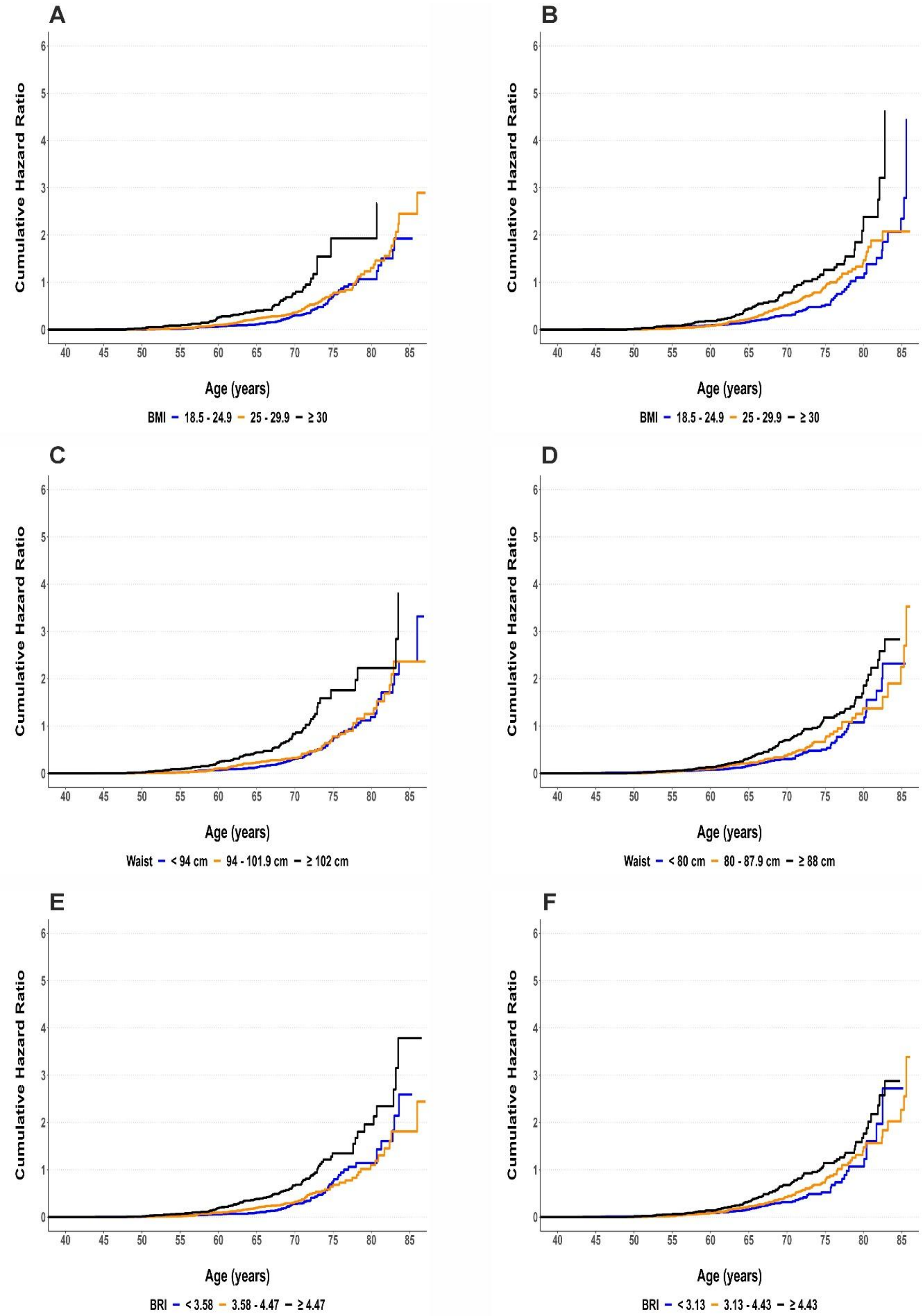

Supplementary figure 3A-3F. Cumulative hazard ratios for multimorbidity (≥4 diseases) by baseline waist circumference, body mass index (BMI), and body roundness index (BRI) categories. Supplementary Figures 3A, 3C, and 3E show data for men and 3B, 3D and 3F for women. Models are adjusted for study baseline age and smoking.

Supplementary Table 2. Hazard ratios for the adjustment variables (age and smoking) in the Cox regression models presented in Table 2.

	≥2 diseases aHR (95% CI)	≥3 diseases aHR (95% CI)	≥4 diseases aHR (95% CI)
**Women**			
**BMI**			
Age	0.96 (0.95–0.98)	0.96 (0.95–0.98)	0.96 (0.94–0.98)
Smoking	1.55 (1.24–1.94)	1.65 (1.28–2.12)	1.73 (1.31–2.29)
**WC**			
Age	0.96 (0.95–0.98)	0.96 (0.94–0.98)	0.96 (0.94–0.98)
Smoking	1.53 (1.22–1.91)	1.60 (1.25–2.05)	1.68 (1.27–2.21)
**BRI**			
Age	0.96 (0.95–0.97)	0.96 (0.94–0.98)	0.96 (0.94–0.98)
Smoking	1.53 (1.22–1.91)	1.60 (1.24–2.05)	1.67 (1.26–2.19)
**Men**			
**BMI**			
Age	0.95 (0.94–0.96)	0.96 (0.94–0.97)	0.96 (0.94–0.97)
Smoking	1.38 (1.12–1.69)	1.71 (1.36–2.14)	1.91 (1.48–2.48)
**WC**			
Age	0.95 (0.93–0.96)	0.95 (0.94–0.97)	0.95 (0.93–0.97)
Smoking	1.28 (1.05–1.57)	1.60 (1.28–2.01)	1.80 (1.39–2.33)
**BRI**			
Age	0.95 (0.93–0.96)	0.95 (0.94–0.97)	0.95 (0.93–0.97)
Smoking	1.33 (1.09–1.63)	1.67 (1.33–2.10)	1.85 (1.43–2.40)

aHR: adjusted hazard ratio; CI: confidence interval; BMI: Body mass index; WC: Waist circumference; BRI: Body roundness index.

Supplementary Table 3. The risk of multimorbidity (≥2 chronic diseases) in a 23-year follow-up by baseline body mass index, waist circumference and body roundness index using Cox regression analysis with alternative adjustment models.

	**Model 1 aHR (95% CI)**	**Model 2 aHR (95% CI)**	**Model 3 aHR (95% CI)**
**Women**			
**Body mass index (kg/m²) at baseline** (Model 1: n=329; Model 2: n=212; Model 3: n=243)			
18.5–24.9	ref.	ref.	ref.
25–29.9	1.28 (0.98–1.67)	1.07 (0.75–1.51)	1.05 (0.76–1.45)
≥30	1.95 (1.38–2.75)	1.82 (1.15–2.89)	1.65 (1.10–2.48)
**Waist circumference (cm) at baseline** (Model 1: n=328; Model 2: n=211; Model 3: n=242)			
<80	ref.	ref.	ref.
80–87.9	1.33 (0.99–1.78)	1.24 (0.85–1.81)	1.25 (0.87–1.79)
≥88	1.46 (1.09–1.96)	1.21 (0.83–1.78)	1.28 (0.91–1.80)
**Body roundness index at baseline** (Model 1: n=328; Model 2: n=211; Model 3: n=242)			
<3.13	ref.	ref.	ref.
3.13–4.43	1.26 (0.94–1.69)	1.17 (0.82–1.69)	1.16 (0.82–1.63)
>4.43	1.60 (1.16–2.19)	1.33 (0.88–2.01)	1.52 (1.05–2.19)
**Men**			
**Body mass index (kg/m²) at baseline** (Model 1: n=259; Model 2: n=182; Model 3: n=207)			
18.5–24.9 (ref)	ref.	ref.	ref.
25–29.9	1.33 (0.99–1.77)	1.50 (1.05–2.15)	1.48 (1.07–2.04)
≥30	2.65 (1.70–4.13)	4.46 (2.48–8.02)	2.50 (1.47–4.25)
**Waist circumference (cm) at baseline** (Model 1: n=258; Model 2: n=181; Model 3: n=206)			
<94	ref.	ref.	ref.
94–101.9	1.24 (0.91–1.69)	1.29 (0.89–1.88)	1.33 (0.94–1.89)
≥102	2.55 (1.78–3.66)	3.52 (2.20–5.64)	2.80 (1.82–4.31)
**Body roundness index at baseline** (Model 1: n=258; Model 2: n=181; Model 3: n=206)			
<3.59	ref.	ref.	ref.
3.59–4.47	1.12 (0.81–1.54)	1.21 (0.82–1.79)	1.17 (0.82–1.67)
>4.47	1.95 (1.39–2.72)	2.11 (1.39–3.20)	1.94 (1.32–2.86)

aHR: adjusted hazard ratio; CI: confidence interval; Ref.: reference category.
Model 1 = adjusted for baseline age and smoking
Model 2 = adjusted for baseline age, smoking, and socioeconomic status
Model 3 = adjusted for baseline age, smoking, and education.
Sample sizes vary across models due to missing covariate data. All analyses were based on participants with available data at the second follow-up (22-year follow-up).

Supplementary Table 4. The risk of complex multimorbidity (≥3 chronic diseases) in a 23-year follow-up by baseline body mass index, waist circumference and body roundness index using Cox regression analysis with alternative adjustment models.

	**Model 1**<br>**aHR (95% CI)**	**Model 2**<br>**aHR (95% CI)**	**Model 3**<br>**aHR (95% CI)**
**Women**			
**Body mass index (kg/m²) at baseline** (Model 1: n=329; Model 2: n=212; Model 3: n=243)			
18.5–24.9	ref.	ref.	ref.
25–29.9	1.30 (0.98–1.74)	1.20 (0.82–1.76)	1.11 (0.78–1.58)
≥30	2.04 (1.41–2.96)	2.02 (1.21–3.38)	1.86 (1.19–2.90)
**Waist circumference (cm) at baseline** (Model 1: n=328; Model 2: n=211; Model 3: n=242)			
<80	ref.	ref.	ref.
80–87.9	1.55 (1.12–2.14)	1.52 (1.00–2.33)	1.41 (0.94–2.11)
≥88	1.78 (1.30–2.45)	1.72 (1.14–2.60)	1.64 (1.13–2.38)
**Body roundness index at baseline** (Model 1: n=328; Model 2: n=211; Model 3: n=242)			
<3.13	ref.	ref.	ref.
3.13–4.43	1.35 (0.98–1.87)	1.27 (0.84–1.90)	1.22 (0.83–1.78)
>4.43	1.72 (1.22–2.42)	1.72 (1.11–2.68)	1.69 (1.14–2.52)
**Men**			
**Body mass index (kg/m²) at baseline** (Model 1: n=259; Model 2: n=182; Model 3: n=207)			
18.5–24.9 (ref)	ref.	ref.	ref.
25–29.9	1.37 (0.99–1.91)	1.51 (1.01–2.27)	1.62 (1.12–2.35)
≥30	3.57 (2.24–5.68)	5.97 (3.28–10.9)	3.55 (2.05–6.15)
**Waist circumference (cm) at baseline** (Model 1: n=258; Model 2: n=181; Model 3: n=206)			
<94	ref.	ref.	ref.
94–101.9	1.25 (0.88–1.78)	1.17 (0.77–1.80)	1.33 (0.90–1.98)
≥102	2.95 (2.01–4.34)	4.09 (2.49–6.74)	3.32 (2.09–5.27)
**Body roundness index at baseline** (Model 1: n=258; Model 2: n=181; Model 3: n=206)			
<3.59	ref.	ref.	ref.
3.59–4.47	1.08 (0.75–1.56)	1.12 (0.72–1.75)	1.08 (0.71–1.62)
>4.47	2.10 (1.46–3.02)	2.19 (1.40–3.43)	2.09 (1.38–3.17)

aHR: adjusted hazard ratio; CI: confidence interval; Ref.: reference category.
Model 1 = adjusted for baseline age and smoking
Model 2 = adjusted for baseline age, smoking, and socioeconomic status
Model 3 = adjusted for baseline age, smoking, and education.
Sample sizes vary across models due to missing covariate data. All analyses were based on participants with available data at the second follow-up (22-year follow-up).

Supplementary Table 5. The risk of complex multimorbidity (≥4 chronic diseases) in a 23-year follow-up by baseline body mass index, waist circumference and body roundness index using Cox regression analysis with alternative adjustment models.

	**Model 1**<br>**aHR (95% CI)**	**Model 2**<br>**aHR (95% CI)**	**Model 3**<br>**aHR (95% CI)**
**Women**			
**Body mass index (kg/m²) at baseline** (Model 1: n=329; Model 2: n=212; Model 3: n=243)			
18.5–24.9	ref.	ref.	ref.
25–29.9	1.30 (0.93–1.82)	1.47 (0.95–2.28)	1.29 (0.86–1.94)
≥30	2.35 (1.57–3.53)	3.01 (1.72–5.27)	2.26 (1.37–3.72)
**Waist circumference (cm) at baseline** (Model 1: n=328; Model 2: n=211; Model 3: n=242)			
<80	ref.	ref.	ref.
80–87.9	1.41 (0.96–2.06)	1.43 (0.86–2.37)	1.16 (0.71–1.89)
≥88	1.89 (1.31–2.71)	2.28 (1.42–3.67)	1.94 (1.27–2.96)
**Body roundness index at baseline** (Model 1: n=328; Model 2: n=211; Model 3: n=242)			
<3.13	ref.	ref.	ref.
3.13–4.43	1.36 (0.93–1.98)	1.30 (0.80–2.10)	1.16 (0.74–1.82)
>4.43	1.93 (1.30–2.84)	2.33 (1.41–3.85)	2.04 (1.30–3.20)
**Men**			
**Body mass index (kg/m²) at baseline** (Model 1: n=259; Model 2: n=182; Model 3: n=207)			
18.5–24.9 (ref)	ref.	ref.	ref.
25–29.9	1.67 (1.14–2.45)	1.64 (1.03–2.63)	1.80 (1.17–2.77)
≥30	3.65 (2.15–6.20)	5.26 (2.76–10.0)	4.09 (2.17–7.72)
**Waist circumference (cm) at baseline** (Model 1: n=258; Model 2: n=181; Model 3: n=206)			
<94	ref.	ref.	ref.
94–101.9	1.17 (0.78–1.75)	1.04 (0.63–1.71)	1.09 (0.69–1.74)
≥102	2.92 (1.94–4.39)	4.31 (2.59–7.19)	3.81 (2.31–6.29)
**Body roundness index at baseline** (Model 1: n=258; Model 2: n=181; Model 3: n=206)			
<3.59	ref.	ref.	ref.
3.59–4.47	1.04 (0.68–1.60)	1.01 (0.61–1.69)	0.90 (0.56–1.46)
>4.47	2.17 (1.45–3.24)	2.15 (1.32–3.52)	2.08 (1.31–3.30)

aHR: adjusted hazard ratio; CI: confidence interval; Ref.: reference category.
Model 1 = adjusted for baseline age and smoking
Model 2 = adjusted for baseline age, smoking, and socioeconomic status
Model 3 = adjusted for baseline age, smoking, and education.
Sample sizes vary across models due to missing covariate data. All analyses were based on participants with available data at the second follow-up (22-year follow-up).

Supplementary Table 6. Sex-specific ROC analysis derived thresholds with optimal sensitivity and specificity for multimorbidity risk based on BMI, WC, and BRI, including AUC (with 95% CI) and Youden's index values.

		Threshold	Sensitivity	Specificity	Youden index	AUC	CI for AUC*
BMI	Men	26.1	0.50	0,75	1.26	0,64	0.57 – 0.71
	Women	26.4	0.47	0.77	1.24	0,64	0.57 – 0.71
Waist (cm)	Men	93.3	0.52	0.73	1.26	0,65	0.58 – 0.72
	Women	76.3	0.79	0.43	1.22	0,65	0.58 – 0.72
BRI	Men	3.70	0.65	0.63	1.28	0,67	0.59 – 0.74
	Women	3.41	0.62	0.66	1.28	0,67	0.60 – 0.74

ROC: receiver operating characteristic, AUC: Area under curve, CI: Confidential interval, BMI: Body mass index, WC: Waist circumference, BRI: Body roundness index

Supplementary table S7. Completed STROBE checklist.

STROBE Statement—checklist of items that should be included in reports of observational studies

	Item No.	Recommendation	Page No.	Relevant text from manuscript
**Title and abstract**	1	(*a*) Indicate the study's design with a commonly used term in the title or the abstract	Title page	See: Title
		(*b*) Provide in the abstract an informative and balanced summary of what was done and what was found	Abstract	See: Abstract
**Introduction**				
Background/rationale	2	Explain the scientific background and rationale for the investigation being reported	pp. 3-4	See: Introduction, 5th paragraph
Objectives	3	State specific objectives, including any prespecified hypotheses	pp. 3-4	See: introduction, 4th paragraph
**Methods**				
Study design	4	Present key elements of study design early in the paper	p. 5	See: Study design
Setting	5	Describe the setting, locations, and relevant dates, including periods of recruitment, exposure, follow-up, and data collection	p. 5	See: Study design and study population
Participants	6	(*a*) *Cohort study*—Give the eligibility criteria, and the sources and methods of selection of participants. Describe methods of follow-up<br>*Case-control study*—Give the eligibility criteria, and the sources and methods of case ascertainment and control selection. Give the rationale for the choice of cases and controls<br>*Cross-sectional study*—Give the eligibility criteria, and the sources and methods of selection of participants	p. 5	See: Study design and study population
		(*b*) *Cohort study*—For matched studies, give matching criteria and number of exposed and	p. 10	See: Table 1 data

		unexposed<br>*Case-control study*—For matched studies, give matching criteria and the number of controls per case		
Variables	7	Clearly define all outcomes, exposures, predictors, potential confounders, and effect modifiers. Give diagnostic criteria, if applicable	pp. 5-7	See: Definitions of obesity and multimorbidity and smoking
Data sources/ measurement	8*	For each variable of interest, give sources of data and details of methods of assessment (measurement). Describe comparability of assessment methods if there is more than one group	pp. 5-7	See: Definitions of obesity and multimorbidity and smoking
Bias	9	Describe any efforts to address potential sources of bias	p. 8	See: Statistical analyses and sensitivity analyses
Study size	10	Explain how the study size was arrived at	p. 5	See: Study population

Continued on next page

Quantitative variables	11	Explain how quantitative variables were handled in the analyses. If applicable, describe which groupings were chosen and why	pp. 5-8	See: Definitions of obesity and multimorbidity and statistical analyses
Statistical methods	12	(*a*) Describe all statistical methods, including those used to control for confounding	p. 8	See: Statistical analyses and sensitivity analyses
		(*b*) Describe any methods used to examine subgroups and interactions	p. 8	See: Statistical analyses and sensitivity analyses
		(*c*) Explain how missing data were addressed	p. 5 and p.8	See: Study population and statistical analyses
		(*d*) *Cohort study*—If applicable, explain how loss to follow-up was addressed<br>*Case-control study*—If applicable, explain how matching of cases and controls was addressed<br>*Cross-sectional study*—If applicable, describe analytical methods taking account of sampling strategy	p. 8	See: Statistical analyses
		(*e*) Describe any sensitivity analyses	p. 8	See: Statistical analyses and sensitivity analyses
**Results**				
Participants	13*	(a) Report numbers of individuals at each stage of study—eg numbers potentially eligible, examined for eligibility, confirmed eligible, included in the study, completing follow-up, and analysed	p. 5	See: Study population and Table 1 data
		(b) Give reasons for non-participation at each stage		
		(c) Consider use of a flow diagram		

Descriptive data	14*	(a) Give characteristics of study participants (eg demographic, clinical, social) and information on exposures and potential confounders	p. 10	See: Table 1 data
		(b) Indicate number of participants with missing data for each variable of interest	p. 10	See: Table 1 data
		(c) *Cohort study*—Summarise follow-up time (eg, average and total amount)	p. 8	See: Statistical analyses
Outcome data	15*	*Cohort study*—Report numbers of outcome events or summary measures over time	p. 11	See: Results
		Case-control study—Report numbers in each exposure category, or summary measures of exposure		
		Cross-sectional study—Report numbers of outcome events or summary measures		
Main results	16	(*a*) Give unadjusted estimates and, if applicable, confounder-adjusted estimates and their precision (eg, 95% confidence interval). Make clear which confounders were adjusted for and why they were included	pp. 11-12	See: Results and Table 2.
		(*b*) Report category boundaries when continuous variables were categorized	pp. 5-7	See: Definitions of obesity and multimorbidity and statistical analyses
		(*c*) If relevant, consider translating estimates of relative risk into absolute risk for a meaningful time period		

Continued on next page

Other analyses	17	Report other analyses done—eg analyses of subgroups and interactions, and sensitivity analyses	p. 15	See: Results, sensitivity analyses
**Discussion**				
Key results	18	Summarise key results with reference to study objectives	p. 16	See: Main results
Limitations	19	Discuss limitations of the study, taking into account sources of potential bias or imprecision. Discuss both direction and magnitude of any potential bias	pp. 18-20	See: Strengths and weaknesses
Interpretation	20	Give a cautious overall interpretation of results considering objectives, limitations, multiplicity of analyses, results from similar studies, and other relevant evidence	pp. 16-21	See: Comparison to existing literature, strengths and weaknesses
Generalisability	21	Discuss the generalisability (external validity) of the study results	pp. 16-17	See Discussion, sensitivity analyses and limitations
**Other information**				
Funding	22	Give the source of funding and the role of the funders for the present study and, if applicable, for the original study on which the present article is based	p. 22	See: Funding statement

*Give information separately for cases and controls in case-control studies and, if applicable, for exposed and unexposed groups in cohort and cross-sectional studies.
